# Supplementary material for: Nanomechanical characterization of quantum interference in a topological insulator nanowire
Source: Nat Commun. 2019 Oct 4;10:4522. doi: 10.1038/s41467-019-12560-4 (PMC6778141; doi:10.1038/s41467-019-12560-4)
Supplement: Supplementary file 1 — Supplementary Information [file 41467_2019_12560_MOESM1_ESM.pdf]

Supplementary Information

Nanomechanical characterization of quantum interference in a topological insulator nanowire

Minjin Kim, Jihwan Kim, Yasen Hou, Dong Yu, Yong-Joo Doh, Bongsoo Kim,

Kun Woo Kim* and Junho Suh*

Supplementary Note 1 | Magneto-conductance for different disorder strengths

The *Methods* section in the main text contains details of conductance calculation. Here, we add conductance maps for different disorder strengths.

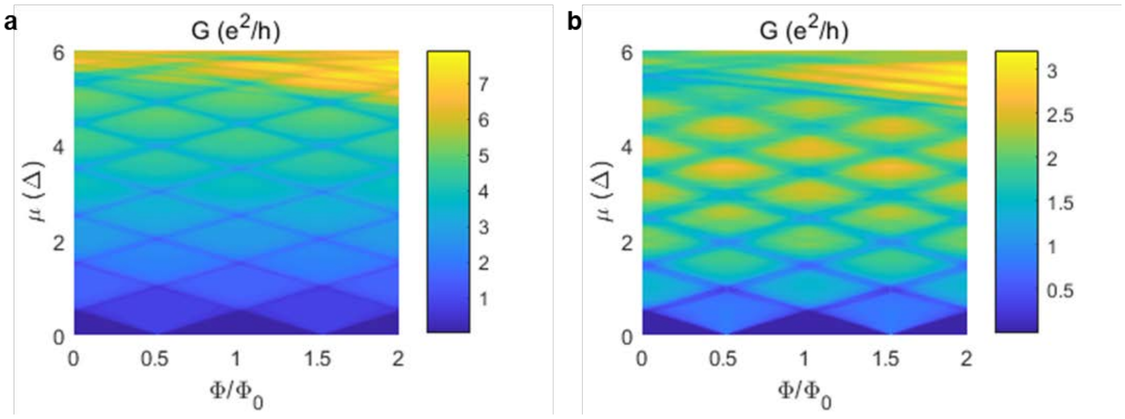

Supplementary Figure 1 | Conductance maps at disorder strengths of (**a**) $W = 1.5\ \Delta$, (**b**) $W = 3.0\ \Delta$.

Without disorder, the band gap is at $\mu = 6.0\ \Delta$, where Δ is the level spacing between neighboring transverse modes at $k = 0$. The Supplementary Figure 1 is conductance maps at disorder strengths of $W = 1.5\ \Delta$ and $3.0\ \Delta$. The horizontal axis is magnetic flux from zero to two quanta. The AB modulation of conductance is clearly seen along the energy (vertical axis), as well as along the magnetic flux. As opposed to a step-like jump of conductance in a clean nanowire, in disordered cases we can see dips develop between conductance plateaus. This indicates that, in the presence of onsite random chemical potential, and near the energy following the diamond-shaped lines, the number of transverse modes engaged in transport fluctuates, thereby causing backscattering. The width of such dips widens for $W = 3.0\ \Delta$.

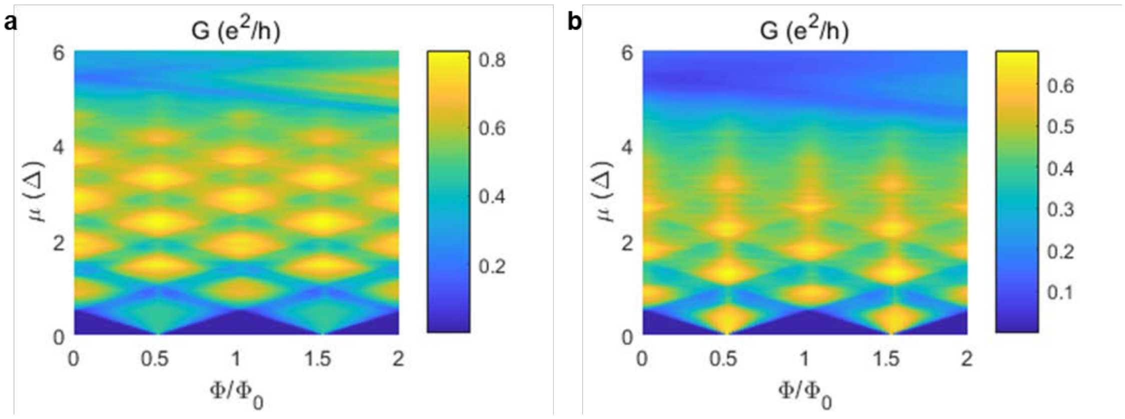

Supplementary Figure 2 | Conductance maps at disorder strengths of (**a**) $W = 4.5\ \Delta$ and (**b**) $W = 6.0\ \Delta$.

Next, the Supplementary Figure 2 is conductance maps for disorder strengths of $W = 4.5\ \Delta$ and $6.0\ \Delta$. Quite remarkably, a feature of weak anti-localization or so-called Altschuler–Aronov–Spivak (AAS) oscillation[1] begins to develop as the crossover from a quasi-1D wire to diffusive 2D transport takes place, when the energy broadening by disorder is larger than the energy spacing between neighboring transverse modes. The period of AAS oscillation along the magnetic flux is $\Phi = \pi$, and it is energy independent. The Supplementary Figure 2b shows clear 'pillars' of conductance along the energy.

For Gaussian-correlated impurities in continuum space, $\langle V(r')V(r)\rangle = K_0 \frac{(\hbar v_F)^2}{2\pi\xi^2} e^{-|r-r'|^2/2\xi^2}$, the elastic linewidth broadening η is given by[2]:

$$\frac{\hbar}{\tau} = \int \frac{dqdr}{4\pi}\left(1 - cos^2\theta_q\right)\delta(k_F - q)\langle V(0)V(r)\rangle e^{iq\cdot r} \ ,$$

$$= \frac{\hbar v_F}{\xi} \frac{2K_0 I_1[(k_F\xi)^2]}{k_F\xi exp[(k_F\xi)^2]} \ ,$$

where k_F is a wavenumber at Femi energy, $\hbar$ is the reduced Planck's constant, $K_0 = \frac{1}{3}{\gamma_0}^2/(\hbar v_F d)^2$ is dimensionless disorder strength, d is mean distance between impurities, and I_1 is a modified Bessel function. We want to compute this quantity for our lattice model. We set onsite random chemical potential on every lattice with uniform distribution $V_n \in$

$[-W, W]$, $H_{\text{dis}} = \sum_{n\in S} \psi_n^\dagger (V_n \Gamma^0) \psi_n$ instead of impurities with Gaussian profiles spread in space. In this case, with the lattice spacing a, $d = a$, $W = \gamma_0 a^2$, $\xi = a$ (spatial resolution), $\Delta = \frac{\hbar v_{\text{F}}}{R}$ (transverse mode energy-level spacing), and $k_{\text{F}}\xi = \frac{E}{\Delta}\frac{a}{R}$. Collecting all,

$$\frac{\hbar}{\tau}\left(\frac{1}{\Delta}\right) = \frac{2}{3}\left(\frac{W}{\Delta}\right)^2 \left(\frac{\Delta}{E}\right) \frac{I_1[(k_{\text{F}}\xi)^2]}{exp[(k_{\text{F}}\xi)^2]} .$$

For example, when $\frac{\hbar}{\tau} = 0.2\ \Delta$, the scattering time is $\tau = 0.7$ ps. Note that the scattering time goes to infinity at the Dirac point as $I_1(x) \sim x$ for $x \ll 1$. Linewidth broadening η for four disorder strengths is plotted Supplementary Figure 3.

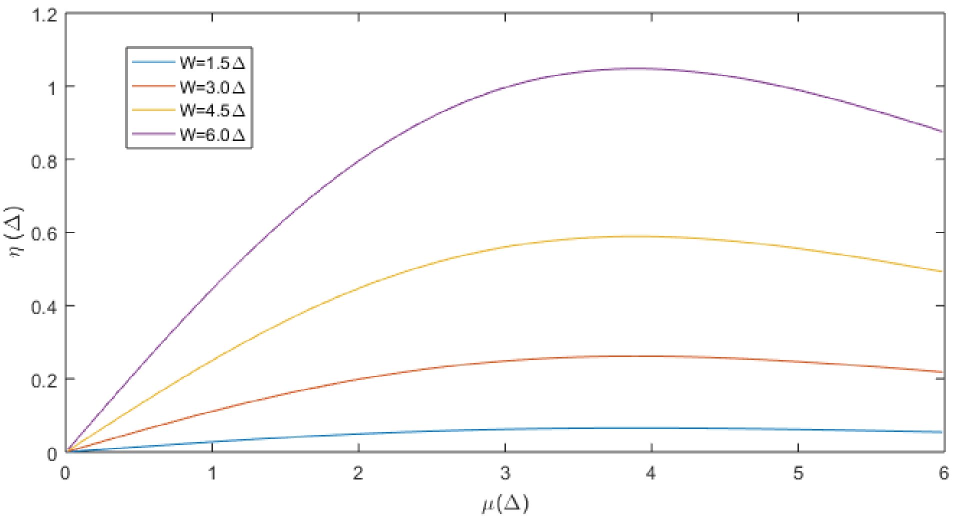

Supplementary Figure 3 | Linewidth broadening η for four disorder strengths of $W = 1.5\ \Delta$, $3.0\ \Delta$, $4.5\ \Delta$, and $6.0\ \Delta$.

For $W = 6.0\ \Delta$, as the energy broadening becomes comparable to the energy level spacing of neighboring transverse modes, the crossover from quasi-1D to diffusive 2D transport can be observed, with our conductance map (Supplementary Figure 2b) clearly showing AAS oscillation as well as AB oscillation with increasing Fermi energy. Lastly, we comment that the energy broadening factor ($\eta = 0.20\ \Delta$) used in our DOS calculation corresponds to an impurity strength between $W = 3.0\ \Delta$ and $W = 4.5\ \Delta$. Even though the

resonant frequency shift map and the conductance map (Supplementary Figure 12) are obtained in different energy windows, the energy broadening used for the former is consistent with the disorder strength used for the latter.

Supplementary Note 2 | Modeling and calculation of geometric capacitance

Multiple experimental observations provided guidance for our numerical modeling: (i) an approximated chemical potential (~24 Δ) estimated from the measurement of total conductance, Fig. 2c; (ii) the periodicity (~5.7 V/Δ) of Aharonov–Bohm (AB) oscillation along the energy, Fig. 2b; (iii) the value of $\ddot{C}_{\mathrm{G}}$ at $V_{\mathrm{g}} = 0$ obtained from the quadratic functional shape of the resonant frequency shift, Fig. 1c; and (iv) the out-of-phase relation between magneto-conductance and resonant frequency shift, Supplementary Figure 12a–b in Supplementary Note 7, which indicates $\Delta f_{\mathrm{I}} < \Delta f_{\mathrm{II}}$. In this section, we devise a geometric capacitance model that satisfies the above conditions.

Supplementary Figure 4 plots the relation between gate voltage and chemical potential ($\mu_0 = 29\ \Delta$ is chosen) according to Kirchhoff's circuit law based on a geometric capacitance of C_{G} = 1.5×10^{-17} F. The oscillation of $\mathrm{d}V_{\mathrm{g}}/d\mu$ (inset) reflects the modulation of the DOS, which is computed with energy broadening $\eta = 0.2\ \Delta$ (see Supplementary Note 4 for details). Near $V_{\mathrm{g}} = -30$ (V), we can see that chemical potential is $\mu\ \sim\ 24\ \Delta$ and $\mathrm{d}V_{\mathrm{g}}/d\mu\ \sim\ 5.5$ (V/Δ), consistent with experimental values.

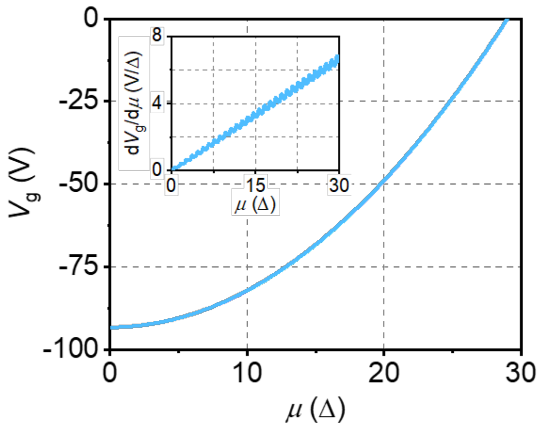

Supplementary Figure 4 | Calculated relation of gate voltage V_{g} and chemical potential μ based on geometric capacitance C_{G} = 1.5×10^{-17} F. Inset: $dV_{\mathrm{g}}/d\mu$ value of about 5.7 V/Δ at a μ range of 23 Δ to 25 Δ. This chemical potential (μ) range corresponds to a V_{g} range of –32 V to –15 V.

Our device setup contains two metals separate in space, namely the Bi_2Se_3 nanowire and the gate plate, so geometric capacitance is the ratio of induced charge and voltage difference between them. We adopt the following two-parallel-plate model:

$$C_{\mathrm{G}}(x) = \frac{\varepsilon_0 A}{d + \alpha x} \ ,$$

where A is effective area, d is distance, x is displacement during the vibration, and α (= 0.523) is a geometrical correction as neither end of the nanowire moves[3]. From this, derivatives are necessary to compute the resonant frequency shift (see Supplementary Note 4 for details):

$$\dot{C}_{\mathrm{G}} = -\alpha \frac{\varepsilon_0 A}{(d + \alpha x)^2} \ , \qquad \ddot{C}_{\mathrm{G}} = 2\alpha^2 \frac{\varepsilon_0 A}{(d + \alpha x)^3} \ .$$

As seen in Fig. 1c, the shift of resonant frequency is nearly symmetric with V_{g}. This overall shape is deduced from the classical circuit model where the change of chemical potential is ignored ($\mu = \mu_0$):

$$\delta U_{\mathrm{ec}} = \ \delta\left(\frac{1}{2} C_{\mathrm{G}} {V_{\mathrm{g}}}^2\right) - \ V_{\mathrm{g}} \delta Q = -\delta\left(\frac{1}{2} C_{\mathrm{G}} {V_{\mathrm{g}}}^2\right) \ .$$

Here, the first term is the charging energy of the capacitor, and the second term is work done to the battery for a fixed gate voltage, $\delta Q = V_{\mathrm{g}} \delta C_{\mathrm{G}}$. Thus, the change in spring constant is $\Delta k = \ddot{U}_{\mathrm{ec}} = -\frac{1}{2} \ddot{C}_{\mathrm{G}} {V_{\mathrm{g}}}^2$, and a parabolic shape with respect to V_{g} naturally appears. From our measurement of resonant frequency shift:

$$\Delta f = \frac{f_0}{2k_0} \Delta k = -\frac{f_0}{4k_0} \ddot{C}_{\mathrm{G}} {V_{\mathrm{g}}}^2 \ ,$$

where $k_0 = (2\pi f_0)^2 m_{\mathrm{eff}}$ and $m_{\mathrm{eff}} = \left(\frac{0.397}{0.523^2}\right) m$, where m = 1.246×10^{-16} kg and the numerical coefficient is again due to the way the nanowire vibrates in its fundamental flexural mode[3]. $\ddot{C}_{\mathrm{G}} =$ 9.91×10^{-4} F/m^2 is estimated from Fig. 1c. Following the two-parallel-plate model, we use d = 174 nm. Since vibration amplitude is much smaller than distance as

estimated in the next paragraph, $x \ll d$, we set x = 0. As a result, $\dot{C}_{\mathrm{G}} = -1.65\times10^{-10}$ F/m. On the other hand, we found C_{G} = 1.5×10^{-17} F is needed to explain 5.7 V/Δ, while the value obtained from integration is $C'_{\mathrm{G}} = \ddot{C}_{\mathrm{G}}\frac{d^2}{2\alpha^2} =$ 5.48×10^{-17} F. Such difference in geometric capacitance may come from an additional capacitance, C_0, which is uninvolved in the mechanical vibration but connected in series to the TI nanowire. Together, $C_{\mathrm{G}} = C'_{\mathrm{G}}C_0/(C'_{\mathrm{G}} + C_0)$ yields the desired geometric capacitance with $C_0 =$ 2.07×10^{-17} F.

A driven mechanical oscillator with natural frequency may have a large oscillating amplitude:

$$x_{\mathrm{RF}} = \frac{F_{\mathrm{RF}}/m_{\mathrm{eff}}}{\sqrt{({\omega_0}^2 - \omega^2)^2 + \omega^2\beta^2}} \ ,$$

where force induced by the voltage source with radio frequency is $F_{\mathrm{RF}} \cong \dot{C}_{\mathrm{G}}V_{\mathrm{g}}V_{\mathrm{RF}}$ and the Q-factor is defined by $Q_{\mathrm{f}} = \sqrt{{\omega_0}^2 - (\beta^2/2)}/\beta \cong \omega_0/\beta$ for a system with a high quality factor (damping rate β is much smaller than ω_0). From Fig. 1c, our device has $Q_{\mathrm{f}} \cong 11900$. We apply $V_{\mathrm{RF}} =$ 0.4 mV, which is much smaller than the gate voltage ($V_{\mathrm{g}} \sim -30$ V) to maintain the small mechanical vibration amplitude $x_{\mathrm{RF}} \approx$ 265 pm, which is to minimize chemical potential change arising during vibration.

With the application of gate voltage, the distance between the nanowire and gate would change by the force associated with the electrostatic energy by

$$F = -\dot{U}_{\mathrm{ec}} - k_0\Delta x_0 = \frac{1}{2}\dot{C}_{\mathrm{G}}{V_{\mathrm{g}}}^2 - k_0\Delta x_0 \ ,$$

which is zero at equilibrium position. We get $\Delta x_0 =$ 0.89 nm. This displacement is much smaller than the nanowire length (1.5 μm) and the distance between the nanowire and gate electrode (d ~ 100 nm). Thus, we assume that the magnetic flux direction is parallel to the current direction along the nanowire, and in the calculation of the resonant frequency shift, we neglect the change of equilibrium position.

Supplementary Note 3 | Density-of-states calculation from the continuum model

Numerical calculation of the DOS of a quasi-1D wire is explained in the *Methods* section of the main text. Here, we add a few more DOS maps to provide more insight.

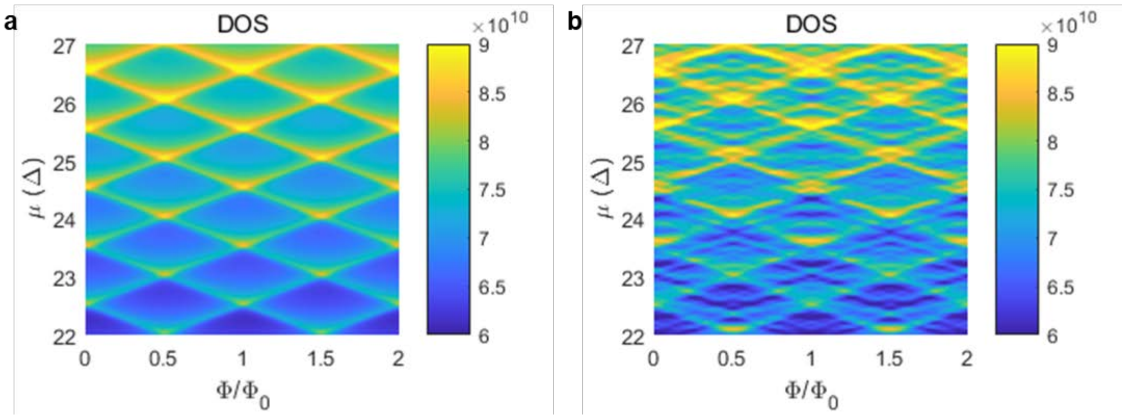

Supplementary Figure 5 | DOS (the number of states per unit length per unit energy eV) of a quasi-1D wire for η = 0.05 Δ when (**a**) wire length is much longer than circumference, $L/(2\pi R) \gg 1$ and (**b**) wire length is three times the circumference, $L/(2\pi R) \cong 3$.

For the energy broadening $\eta = 0.05\ \Delta$ used in Fig. 1f, DOS maps are generated in the domain of magnetic flux and chemical potential. When wire length is much longer than circumference, $L/(2\pi R) \gg 1$ (Supplementary Figure 5a), the DOS shows peaks whenever a new transverse mode is encountered, as the band bottom of a quasi-1D mode has a diverging DOS ($\sim 1/v_\mathrm{F}$). The Supplementary Figure 5b reflects the DOS of the actual dimension ratio, $L/(2\pi R) \cong 3$. On top of the diamond-shaped lines, there are additional modulations of the DOS due to the discretization of eigenmodes along the direction of current propagation, $\Delta' \cong \hbar v_\mathrm{F}/L = \Delta/3$. While conductance is dependent on the number of transverse modes, mechanical resonance shift is influenced by all details of the DOS. While this may complicate characterization of the nanowire, it also provides more information on microscopic details.

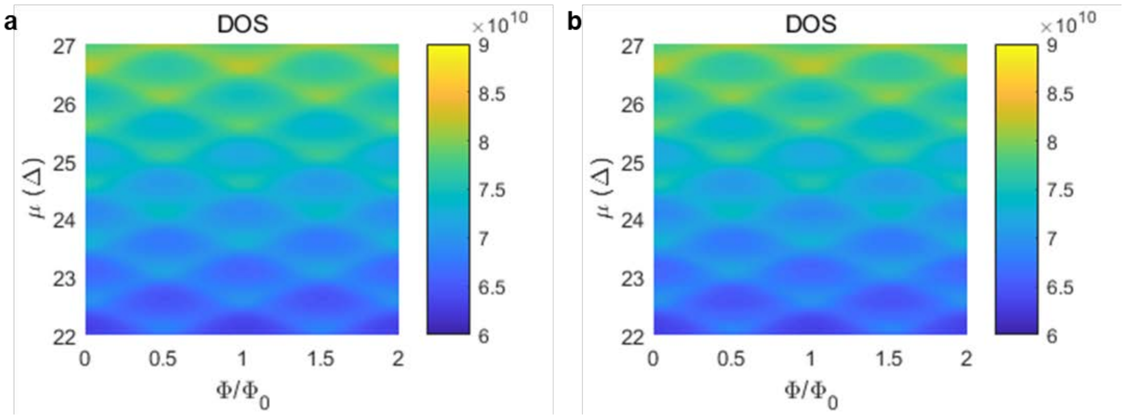

Supplementary Figure 6 | DOS (the number of states per unit length per unit energy eV) of of a quasi-1D wire for η = 0.20 Δ when (**a**) wire length is much longer than circumference, $L/(2\pi R) \gg 1$ and (**b**) wire length is three times the circumference, $L/(2\pi R) \cong 3$.

We present DOS maps for $\eta = 0.20\ \Delta$ (Supplementary Figure 6) for the two geometries used for calculation in the main text. Here, modulation of the DOS is smoothed out, and detailed features of the eigenmodes for the $L/(2\pi R) = 3$ case are significantly reduced. The quantum capacitance is

$$\mathrm{C}_Q = Le^2\nu(E) \cong (1.5\times10^{-6})\times(1.6\times10^{-19})^2\left(\frac{50}{\hbar v_\mathrm{F}}\right) = 3.6\times10^{-14}\ \mathrm{F},$$

where $\nu(\mu \cong 25\Delta)$ is approximated to the DOS of fifty 1D wire with Fermi velocity[4] $v_\mathrm{F} =$ 5×10^5 m/s. As claimed in the manuscript, in the energy window of our measurement the quantum capacitance is ~1000 times larger than the geometric capacitance.

Supplementary Note 4 | Calculation of mechanical resonant frequency shift

To compute the shift in the mechanical resonant frequency of a TI nanowire, we need to know the change in total energy caused by the flexural mode:

$$\delta U_{\mathrm{tot}} = \delta\left(\frac{1}{2}m\dot{x}^2 + \frac{1}{2}k_0 x^2\right) + \delta U_{\mathrm{ec}},$$

$$\delta U_{\mathrm{ec}} = \delta\left(\frac{Q^2}{2C_{\mathrm{G}}}\right) - V_{\mathrm{g}}\delta Q + \delta\left(Q\frac{\mu-\mu_0}{e}\right),$$

where in the first line, total energy is the sum of mechanical energy and electrostatic energy δU_{ec}, and in the second line, δU_{ec} contains three circuit elements: charging energy of the geometrical capacitor, work done to the gate battery, and charging energy of the TI nanowire. Note that the last term cannot be replaced by $\delta\left(Q^2/2C_{\mathrm{Q}}\right)$ as quantum capacitance is a nonlinear function of induced charge. Since our measurements are made by varying gate voltage and magnetic field, hereafter we focus on the resonant frequency shift induced by changes in electrostatic energy.

Our goal is to simply compute the second derivative of U_{ec} with respect to *x*. Several quantities are dependent on *x*. First, as discussed in Supplementary Note 2, geometric capacitance is position-dependent. For a fixed gate voltage, induced charge Q is position-dependent and so is the resulting change of chemical potential μ. Two relations are helpful to simplify further calculation; one is Kirchhoff's law as shown in the main text, and the other is its derivative for a fixed gate voltage:

$$\frac{\partial Q}{\partial x} = \frac{C_{\mathrm{Q}}}{C_{\mathrm{G}}}\frac{Q}{C_{\mathrm{G}} + C_{\mathrm{Q}}}\frac{\partial C_{\mathrm{G}}}{\partial x},$$

where change in chemical potential is expressed in terms of induced charge and quantum capacitance (the DOS) by:

$$\frac{1}{e}\frac{\partial \mu}{\partial x} = \frac{1}{C_Q}\frac{\partial Q}{\partial x},$$

where $C_{\rm Q} = e^2 L\nu$ and $\nu = \delta(Q/Le)/\delta\mu$. Electrostatic energy is first simplified by Kirchhoff's law:

$$\frac{dU_{\rm ec}}{dx} = -\frac{Q^2}{2{C_{\rm G}}^2}\frac{\partial C_{\rm G}}{\partial x} + \frac{Q}{C_Q}\frac{\partial Q}{\partial x} ,$$

$$= -\frac{Q^2}{2{C_{\rm G}}^2}\frac{\partial C_{\rm G}}{\partial x} + \frac{1}{C_{\rm G}}\frac{Q^2}{C_{\rm G} + C_{\rm Q}}\frac{\partial C_{\rm G}}{\partial x} .$$

By taking one more derivative, we obtain the desired result:

$$\frac{d^2U_{\rm ec}}{dx^2} = \frac{1}{{C_{\rm G}}^2}\left[\frac{2C_{\rm Q} - C_{\rm G}}{(C_{\rm G} + C_{\rm Q})^2}{\dot{C}_{\rm G}}^2 + \frac{C_{\rm G} - C_{\rm Q}}{2(C_{\rm G} + C_{\rm Q})}\ddot{C}_{\rm G}\right]Q^2 + \frac{1}{2}\left(\frac{\dot{C}_{\rm G}}{C_{\rm G}}\right)^2 e\frac{\partial}{\partial\mu}\left(\frac{1}{(C_{\rm G} + C_{\rm Q})^2}\right)Q^3 ,$$

$$= k_{\rm I} + k_{\rm II} ,$$

where the dots indicate the derivatives with respect to displacement *x*. So far, no approximation has been made except for the electrostatic condition of our circuit, assuming that mechanical vibration is much slower than the time required for electrical equilibration. The above expression is used for Fig. 3. Since our device has $C_{\rm G} \ll C_{\rm Q}$ and ${\dot{C}_{\rm G}}^2 \cong C_{\rm G}\ddot{C}_{\rm G}$, the expression is simplified to the one shown in the main text. In Supplementary Figure 7, two contributions of resonant frequency shift $\Delta f_{\rm I,II}$ are plotted separately to show their qualitative difference in modulation and scaling over energy.

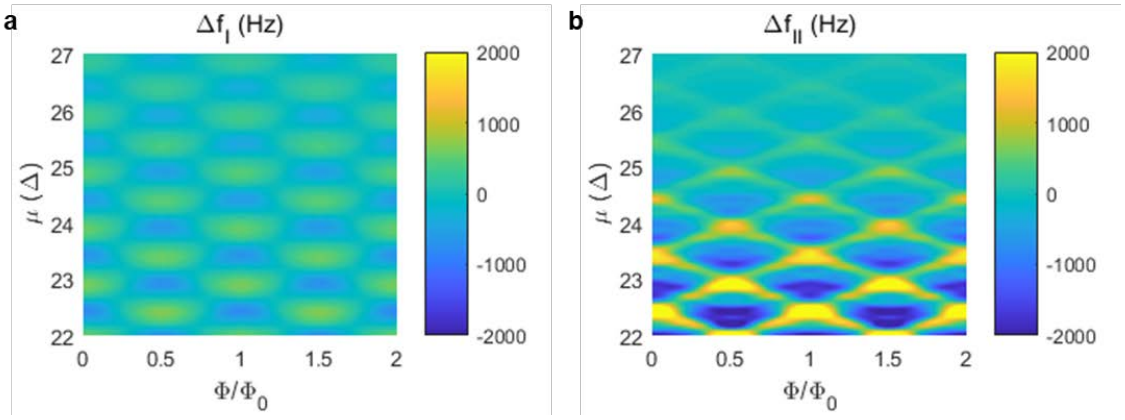

Supplementary Figure 7 | Mechanical resonant frequency shifts (Δf_0) as a result of DOS oscillation via quantum capacitance effects.

Supplementary Note 5 | Characterization of the Bi_2Se_3 nanowire

Bi_2Se_3 nanowire dimensions

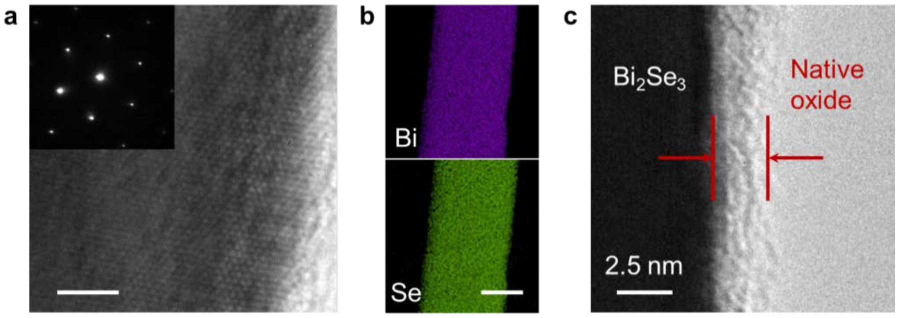

Supplementary Figure 8 | Transmission electron microscope characterization of Bi_2Se_3 nanowire. **a,** High-resolution transmission electron microscope (TEM) image and (inset) selected area electron diffraction pattern of the single-crystalline Bi_2Se_3 nanowire. Scale bar, 2 nm. **b,** Energy-dispersive X-ray spectroscopy elemental maps showing homogeneous atomic distribution of Bi (violet) and Se (green). Scale bar, 100 nm. **c,** TEM image of the Bi_2Se_3 nanowire indicating a 2.5-nm-thick native oxide layer is present on its surface. Cross-sectional area S is calculated by S = (width – 2×native oxide layer thickness) × (thickness – 2×native oxide layer thickness). With a native oxide thickness of 2.5 nm, the nanowire in Device 1 has dimensions of width = 105 nm and thickness = 116 nm with S = 1.11×10^4 nm^2. The ΔB deduced from the cross-sectional area S of Device 1 is 0.37 T, which is given by $\Delta B = \Phi_0/S$ ($\Phi_0 = h/e$, h is Planck's constant, and e is the elementary charge). The effective cross-section is $\Phi_0/\Delta B$ = 1.04×10^4 nm^2, which is slightly smaller than the actual value possibly due to the finite penetration depth of boundary states.

Gate response and Dirac point

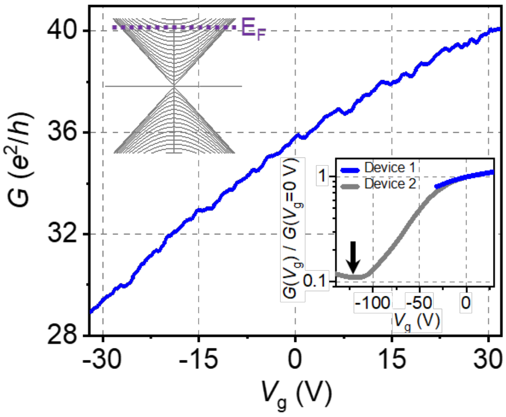

Supplementary Figure 9 | Conductance vs. gate voltage in Device 1. Inset: From comparison with another device of identical geometry (Device 2), the Dirac point is estimated to be at $V_g \approx -113$ V.

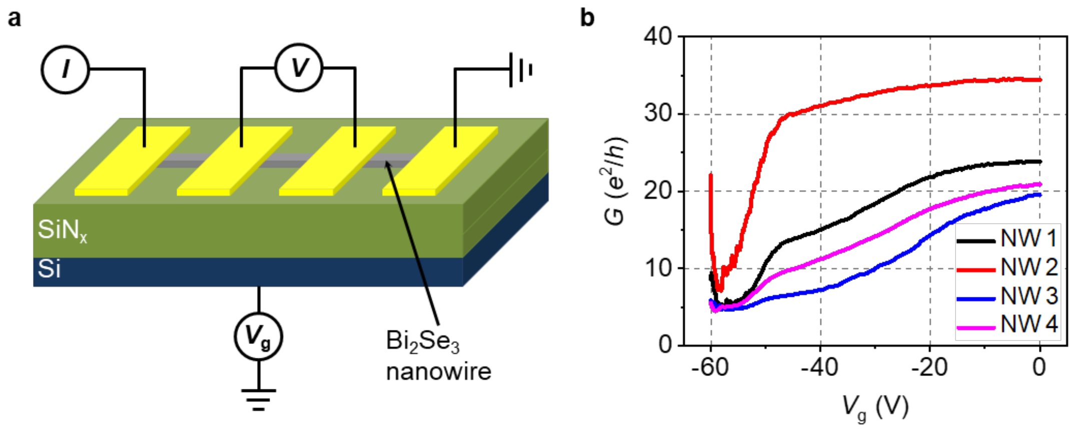

Supplementary Figure 10 | Dirac point of Bi_2Se_3 nanowire. **a,** Conductance measurement schematic showing the Bi_2Se_3 nanowire on a SiN_x/Si substrate with back-gate voltage V_g. **b,** Nanowire (NW) conductance G response to V_g. The four different nanowires exhibit Dirac points at V_g = –57.6 V (NW 1), –58.1 V (NW 2), –57.0 V (NW 3), and –59.1 V (NW 4).

In Supplementary Figure 9, the nanowire conductance of Device 1 decreases with decreasing gate voltage, showing n-type semiconductor behavior. Because Device 1 was destroyed during gating, its Dirac point (V_g = –113 V) was estimated from another similarly fabricated nanowire (Device 2). Even though two nanowires may have a different circumference and impurity concentration, their intrinsic chemical potential μ_0 would be close to each other as it is determined by average chemical composition. As such, the gate voltage required to bring the chemical potential all the way down to the Dirac point is similar for each nanowire, because the DOS per unit length and the induced charge are both linearly proportional to nanowire circumference. This argument is supported by additional measurements of four more nanowires that are in contact with the substrate instead of being suspended (Supplementary Figure 10a). The gate voltage at the Dirac point is different (V_g ~ –58 V) on account of the different geometry, but it is consistent among all four nanowires (Supplementary Figure 10b). It is interesting to note that the wires have different total conductance even though their intrinsic chemical potentials are supposedly the same. This might be due to differences in device-specific impurity concentration; while the amount of

induced charge (integration of the DOS) is less sensitive to impurities, the total conductance could be significantly reduced (see Supplementary Note 1 for conductance calculations for different disorder strengths).

Supplementary Note 6 | Mechanical resonant frequency shift measurement

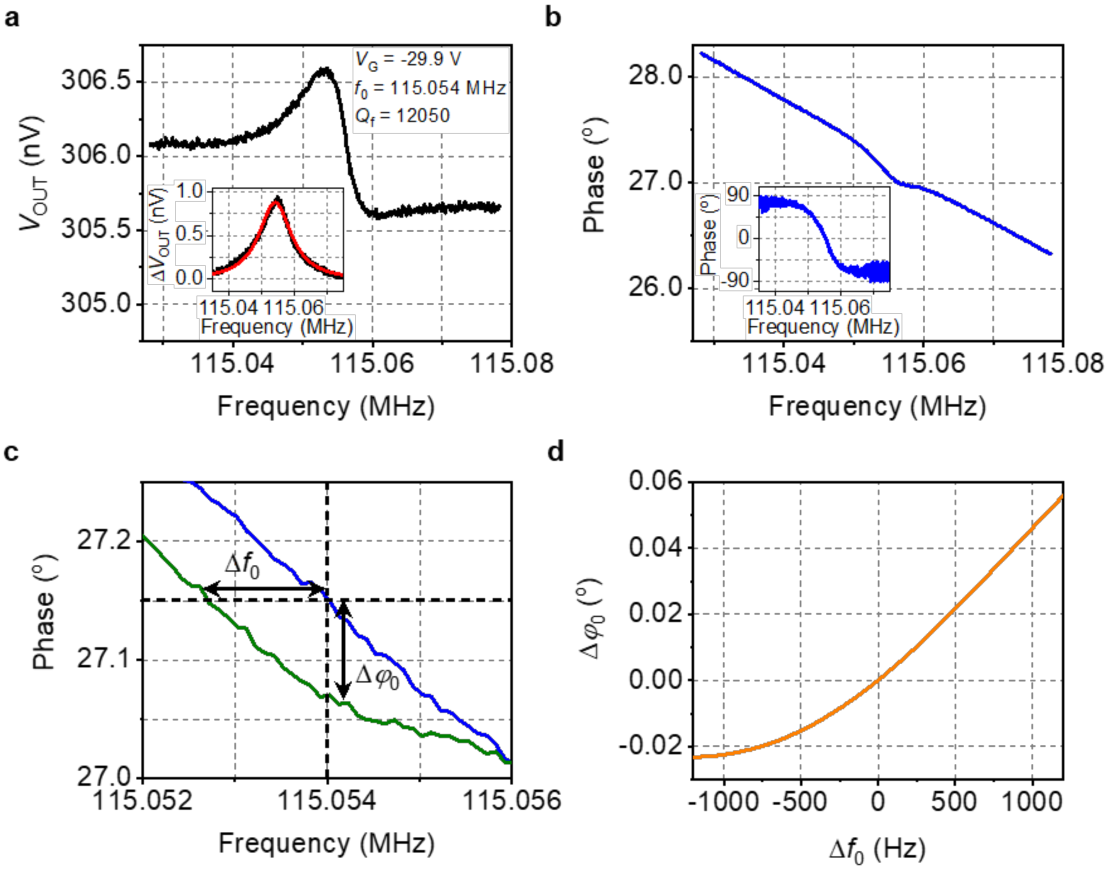

Supplementary Figure 11 | Mechanical resonant frequency shift measurement. **a,** Amplitude response of mechanical resonance. Inset: Amplitude response after subtracting background response. The solid red line is a Lorentzian fit with resonance frequency f_0 and quality factor Q_f. **b,** Phase response of mechanical resonance. Inset: Phase response after subtracting background response. **c,** Phase response change (e.g. from blue to green) when f_0 is modulated by Δf_0 ($\ll f_0/Q_f$), and the phase at f_0 shifts by $\Delta\varphi_0$. **d,** Extracted relation between Δf_0 and $\Delta\varphi_0$.

From our device, we obtain electric signal V_{OUT} oscillating at the frequency of applied radio-frequency voltage V_{RF}, but with a phase difference. The amplitude and phase of V_{OUT}

is plotted in Supplementary Figure 11a–b, respectively. A typical mechanical resonant frequency f_0 is approximately 115 MHz and quality factor Q_f is $\sim 1.2 \times 10^4$. After subtracting the background, a Lorentzian function of amplitude near the resonance and a 180° change of phase are plotted in the insets. In Supplementary Figure 11c, the resonant frequency shift induced by a threading magnetic field and corresponding phase shift at the resonant frequency are shown. Measurements of the phase shifts for a given V_RF and V_g were made by varying the magnetic field. The frequency shift is obtained based on the relation plotted in Supplementary Figure 11d.

Supplementary Note 7 | Correlation between conductance and mechanical resonant frequency shift

Our TI nanowire device setup allows for the simultaneous measurements of electrical conductance and mechanical resonant frequency, with the observation of strong correlation between the two independent signals confirming their common origin: AB oscillation. For two representative magnetic fluxes $\Phi = 1.5\ \Phi_0$ and 2 Φ_0, conductance modulation ΔG and resonant frequency shift Δf_0 with respect to gate voltage are shown in Supplementary Figure 12a–b. We note that the resonant frequency shift is close to being out of phase with conductance modulation in the range of V_g = -32 V ~ -26 V. The observed out of phase correlation is successfully reconstructed by a quasi-1D wire model of a gapless Dirac fermion (Supplementary Figure 12c–f). To compute magneto-conductance, we employ the Landauer–Buttiker formalism for a 3D TI lattice model maintaining the aspect ratio of our nanowire[5,6]. We simulate conductance at the chemical potential in our device by introducing a disorder strength that yields a scattering time close to the one in the nanowire (see Supplementary Note 1 for details). In this way, the observed AB oscillation of conductance is reproduced reasonably well (Supplementary Figure 12d–f). For the modulation of mechanical resonant frequency shift, we employ the quasi-1D model of a Dirac fermion with complete eigenenergy information for a given magnetic flux from equation (3) in the manuscript. Using a scattering time consistent with the disorder strength used in the magneto-conductance calculation, we first obtain the DOS from the Green's function and then compute the shift of resonant frequency according to equation (5) in the manuscript. The calculated frequency modulation successfully explains the experimental data in the range of chemical potential (Supplementary Figure 12c,e,f). Most importantly, the out-of-phase relation between conductance and mechanical resonant frequency shift in Supplementary Figure 12a–b is

clearly reproduced in our model calculations in Supplementary Figure 12e–f, confirming the nature of the TI surface states.

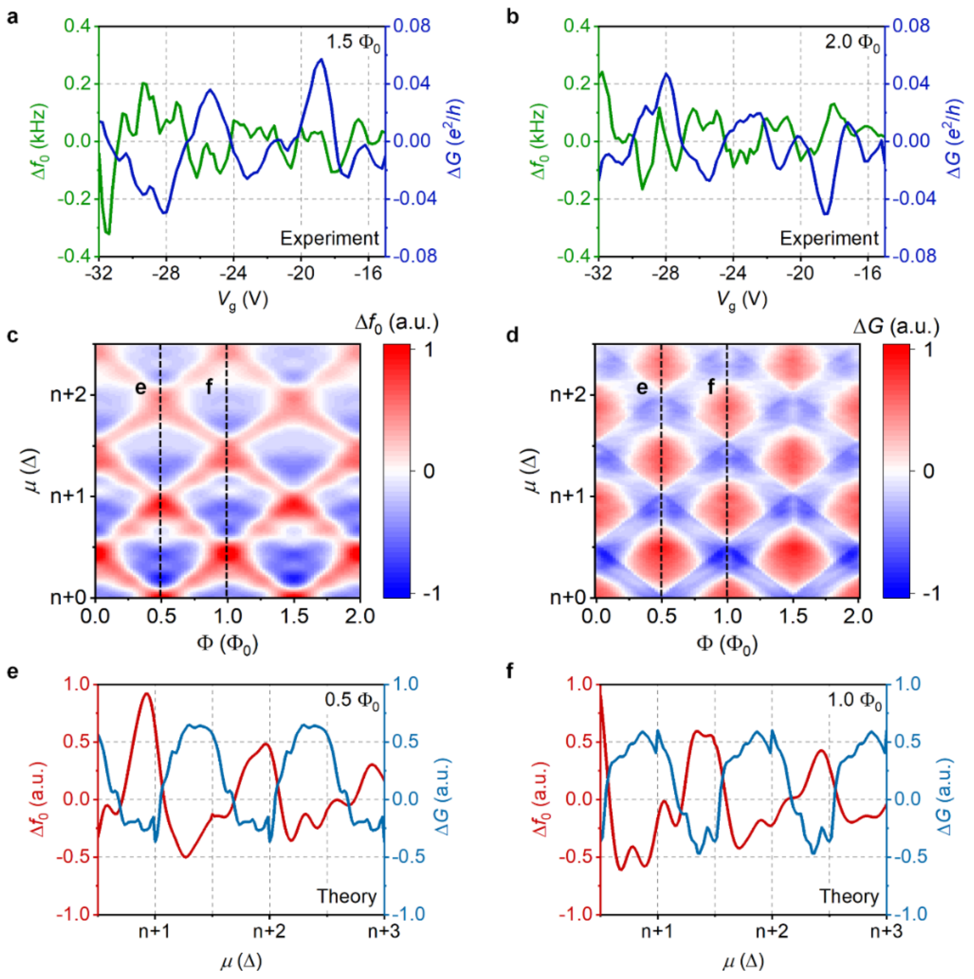

Supplementary Figure 12 | AB oscillations in the mechanical resonant frequency and conductance of a TI nanowire. **a–b**, Measured mechanical resonant frequency shift Δf_0 and conductance modulation ΔG as a function of gate voltage V_g at half-integer flux quanta and integer flux quanta, showing an out-of-phase relation. **c–d**, Model calculations of Δf_0 and ΔG plotted as a function of chemical potential μ and magnetic flux Φ. **e–f**, Calculated Δf_0 and ΔG at half-integer and integer flux quanta conditions demonstrating an out-of-phase relation, plotted from the corresponding vertical cuts in (**c**) and (**d**).

Supplementary Note 8 | Correspondence between the experimental data and simulation maps of mechanical resonant frequency shifts

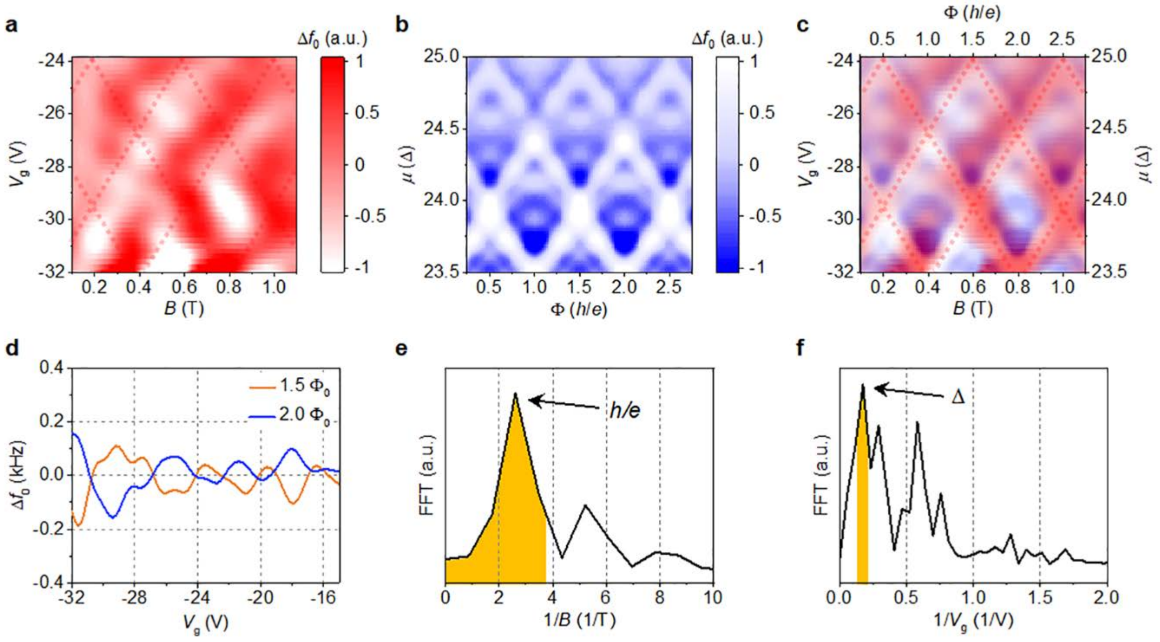

Supplementary Figure 13 | Correspondence between experiment and simulation. **a–b**, Normalized 2D experimental data (**a**) and simulation color map (**b**) of mechanical resonant frequency shifts. **c**, Overlaid plot of experimental data in (**a**) with the simulation color map in (**b**). **d**, Mechanical resonant frequency shift as a function of gate voltages at half-integer and integer flux quanta showing out-of-phase relation. **e–f**, The Fourier component slower than 1.5 *h/e* period in flux (**e**) and the Fourier components within 20 % window about the major oscillation Δ period in energy (**f**) are extracted to plot the experimental data in (**a**) and (**d**).

We compare the 2D experimental data and simulation maps of mechanical resonant frequency shifts in Supplementary Figure 13. The repeating diamond pattern is shown in both maps (Supplementary Figure 13a–b) and matches each other as shown in Supplementary Figure 13c. To compare the pattern, we extract the selected Fourier component (Supplementary Figure 13e–f) in the experimental data and normalize the amplitude in both of the experimental data and simulation map. The mechanical resonant frequency shift versus V_g curves (Supplementary Figure 13d) for integer and half-integer flux quanta also show clear out-of-phase relation with each other after extracting the selected Fourier component (Supplementary Figure 13e–f).

Supplementary Note 9 | Estimate of spin-orbit coupling effect associated with nanomechanical motion

We examine here the possibility of having a Zeeman-type spin splitting by the threaded magnetic field B (~1 T) into the nanowire. We find that, associated with the motion of the resonator, which is driven by the radio-frequency AC voltage source, spin polarization arises on the sides of the nanowire, and that the polarization can couple to the external magnetic field. However, the effect turns out small enough to be neglected. In short, it originates from the interplay between the external AC electric field and the relaxation of helical electronic states. The detailed calculation is as follows.

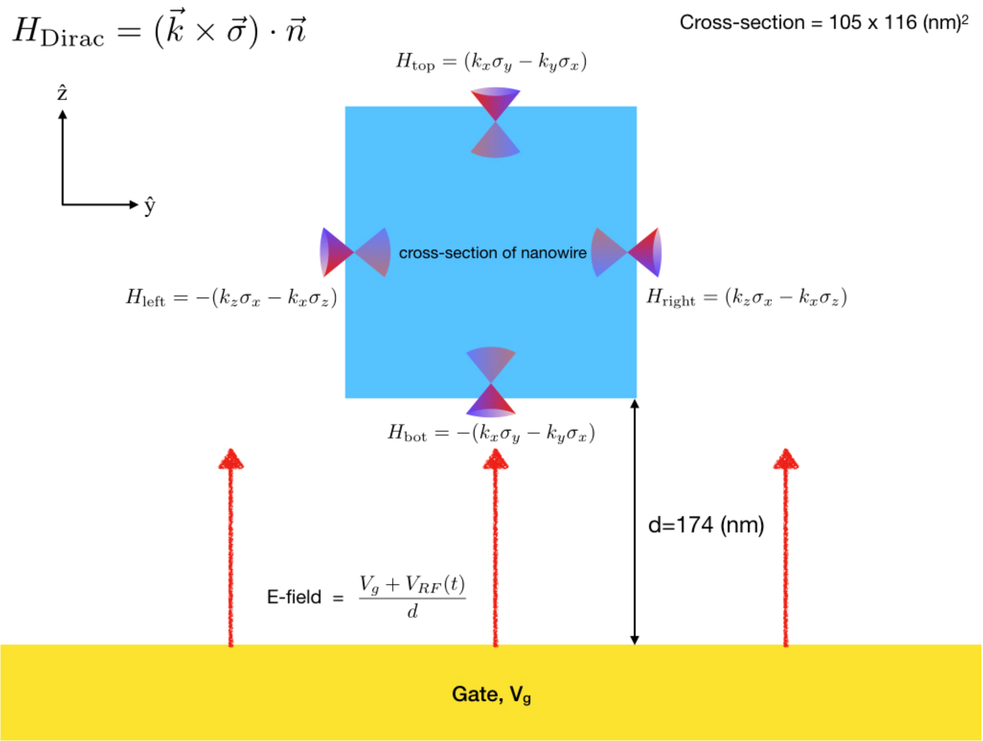

Supplementary Figure 14 | Schematic representation of the topological insulator nanowire device.

In Supplementary Figure 14, the direction of the external magnetic field is out of plane, $\vec{B} = B\hat{x}$. The effective Hamiltonian of Dirac surface states is specified assuming that the penetration depth into the bulk is smaller than the size of nanowire. A time-dependent electric

field is applied from the gate to the nanowire direction, $E_{\mathrm{z}}(t) = V_{\mathrm{RF}}\sin(2\pi f_{\mathrm{RF}}t)/d$ with frequency $f_{\mathrm{RF}} =$ 115 MHz and $V_{\mathrm{RF}} =$ 0.4 mV. On the other hand, the relaxation time of electrons is τ ~1 ps. This implies that the effective external electric field is reduced as

$$E_{\mathrm{eff}}(t) \approx \tau \frac{dE_{\mathrm{z}}(t)}{dt} = 2\pi f_{\mathrm{RF}}\tau \frac{V_{\mathrm{RF}}\cos(2\pi f_{\mathrm{RF}}t)}{d},$$

due to screening by electrons. Note that the vibration amplitude of the nanowire (~265 pm) is negligible compared to d.

The effective electric field drives the electron population out of equilibrium. According to the Boltzmann equation:

$$\begin{aligned}\delta f_{k_z} &= f_{k_z} - f_0 \\ &= e\tau E_{\mathrm{eff}} \frac{df_{k_z}}{dp_z} \approx e\tau E_{\mathrm{eff}} v_F \frac{df_{k_z}}{dE} \approx e\tau \left(2\pi f_{\mathrm{RF}}\tau \frac{V_{\mathrm{RF}}}{d}\right) v_F \frac{1}{kT} \\ &= 0.329\end{aligned}$$

where T = 50 mK and $v_{\mathrm{F}} =$ 5 × 10^5 m/s. In other words, on the right surface of the nanowire (see figure), there are a greater number of electrons going upward ($\hat{z}$) than going downward (–$\hat{z}$). Because Dirac surface states are helical, this naturally makes net spin polarization S_x, which in turn causes a spin-dependent energy shifting by the external magnetic field. Note that on the left surface, the effective surface Hamiltonian is $H_{\mathrm{left}} = -H_{\mathrm{right}}$, and thus the opposite spin polarization and the total energy do not change by the Zeeman splitting.

The energy scale of the Zeeman effect under B = 1 T,

$$\Delta H_{\mathrm{Zeeman}} = \frac{\mu_B g_s}{\hbar}\mathbf{S}\cdot\mathbf{B} \approx 0.058 \text{ meV}.$$

Therefore, the energy shift due to the time-dependent electric field is $\Delta E_{\mathrm{Zeeman}} \approx$ 0.329 × 0.058 meV = 0.019 meV, which is more than two orders of magnitude smaller than the energy level spacing between neighboring transverse modes, $\Delta =$ 4.7 meV. As a result, we

conclude that spin polarization along the magnetic field direction ($\hat{x}$) would appear during the relaxation of electrons in the presence of an AC external electric field, but its influence on our TI nanowire is negligibly small.

Supplementary References

1. Altshuler, B. L., Aronov, A. G., Spivak, B. Z. The Aaronov-Bohm effect in disordered conductors. *JETP Lett.* **33**, 94 (1981).
2. Adam, S., Brouwer, P. W. & Das Sarma, S. Crossover from quantum to Boltzmann transport in graphene. *Phys. Rev. B* **79**, 201404 (2009).
3. LaHaye, M. D. The radio-frequency single-electron transistor displacement detector. (The University of Maryland, 2005).
4. Zhang, H. *et al.* Topological insulators in Bi_2Se_3, Bi_2Te_3 and Sb_2Te_3 with a single Dirac cone on the surface. *Nat. Phys.* **5**, 438–442 (2009).
5. Büttiker, M. Absence of backscattering in the quantum Hall effect in multiprobe conductors. *Phys. Rev. B* **38**, 9375 (1988).
6. Weeks, W. C. A study of topological insulators in two and three dimensions. (University of British Columbia, 2012).
